# Antimalarial, antiplasmodial and analgesic activities of root extract of *Alchornea laxiflora*

**DOI:** 10.1080/13880209.2017.1285947

**Published:** 2017-02-09

**Authors:** Jude E. Okokon, Nkemnele Bensella Augustine, Dinesh Mohanakrishnan

**Affiliations:** a Department of Pharmacology and Toxicology, Faculty of Pharmacy, University of Uyo, Uyo, Nigeria;; b Malaria Research Laboratory, International Centre for Genetic Engineering and Biotechnology, Aruna Asaf Ali Marg, New Delhi, India

**Keywords:** Antinociceptive, malaria, *Plasmodium falciparum*, *P. berghei*

## Abstract

**Context:***Alchornea laxiflora* (Benth.) Pax. & Hoffman (Euphorbiaceae) root decoctions are traditionally used in the treatment of malaria and pain in Nigeria.

**Objective:** To assess the antimalarial, antiplasmodial and analgesic potentials of root extract and fractions against malarial infections and chemically-induced pains.

**Material and methods:** The root extract and fractions of *Alchornea laxiflora* were investigated for antimalarial activity against *Plasmodium berghei* infection in mice, antiplasmodial activity against chloroquine sensitive (Pf 3D7) and resistant (Pf INDO) strains of *Plasmodium falciparum* using SYBR green assay method and analgesic activity against experimentally-induced pain models. Acute toxicity study of the extract, cytotoxic activity against HeLa cells and GCMS analysis of the active fraction were carried out.

**Results:** The root extract (75–225 mg/kg, p.o.) with LD_50_ of 748.33 mg/kg exerted significant (*p* < 0.05–0.001) antimalarial activity against *P. berghei* infection in suppressive, prophylactive and curative tests. The root extract and fractions also exerted moderate activity against chloroquine sensitive (Pf 3D7) and resistant (Pf INDO) strains of *P. falciparum* with the ethyl acetate fraction exerting the highest activity with IC_50_ value of 38.44 ± 0.89 μg/mL (Pf 3D7) and 40.17 ± 0.78 μg/mL (Pf INDO). The crude extract was not cytotoxic to HeLa cells with LC_50_ value >100 μg/mL. The crude extract and ethyl acetate fraction exerted significant (*p* < 0.05–0.001) analgesic activity in all pain models used.

**Discussion and conclusions:** These results suggest that the root extract/fractions of *A. laxiflora* possess antimalarial, antiplasmodial and analgesic potentials and these justify its use in ethnomedicine to treat malaria and pain.

## Introduction

*Alchornea laxiflora* (Benth.) Pax. & Hoffman (Euphorbiaceae) is a deciduous shrub, about 6–10 m high. It grows in most areas of Africa including Nigeria, DR Congo, Ethiopia and throughout East Africa to Zimbabwe (Burkill 1994). *Alchornea laxiflora* is called ‘Opoto and Nwariwa’ respectively among the Yoruba and Ibibio tribes in Nigeria. The root decoctions are used by the Ibibios to treat malaria while leaf infusion is used in folklore medicine by the Yorubas as malarial remedy (Adeloye et al. 2005). The leave decoctions are usually used to treat inflammation and infectious diseases (Ogundipe et al. 2001). Oladunmoye and Kehinde (2011) reported the use of *A. laxiflora* among the Yoruba tribe of southwestern Nigeria for the treatment of poliomyelitis, and measles. Biological activities reported on the leaf include; antioxidant (Farombi et al. 2003; Oloyede et al. 2010), antibacterial and antifungal activities against *Staphylococcus aureus*, *Escherichia coli*, *Bacillus subtilis*, *Pseudomonas aeruginosa,* and fungal species (Oloyede et al. 2010; Akinpelu et al. 2015), hepatoprotective activity (Oloyede et al. 2011), antianaemic activity (Oladiji et al. 2014), antitoxicity, anticonvulsant and sedative effects (Esosa et al. 2013). The leaf extract of *Alchornea laxiflora* has been reported to contain alkaloids, saponins, tannins, phlobatannins, flavonoids and cardiac glycosides, among others (Oloyede et al. 2010; Oladiji et al. 2014). Flavonoids such as quercetin, quercetrin, rutin, taxifolin and quercetin 3, 4-diacetate (Ogundipe et al. 2001; Adeloye et al. 2005; Oloyede et al. 2011), quercetin-3-*O*-I^2^-d-glucopyranoside and quercetin 3, 7, 3′, 4′-tetrasulphate (Oloyede et al. 2011) have been isolated from the ethyl acetate leaf fraction of the plant. Terpenoid compounds were reported to be present in the root extract of *A. laxiflora* (Farombi et al. 2003).

Scientific information regarding the antiplasmodial activity of *A. laxiflora* root is not available. In this investigation, we report the analgesic, *in vivo* antimalarial and *in vitro* antiplasmodial activities of the ethanol root extract and fractions of *Alchornea laxiflora* against *Plasmodium berghei* and chloroquine sensitive and resistant strains of *Plasmodium falciparum* to confirm the folkloric claim of its usefulness in the treatment of malaria traditionally.

## Materials and methods

### Drugs

Chloroquine diphosphate and artemisinin used in this study were from Sigma-Aldrich, Darmstadt, Germany.

### Animals

The animals (Swiss albino mice) of either sex were used for these experiments. Four-week-old mice (16–20 g) were used for malaria study, while 2-month-old mice (25–28 g) were used in the analgesic study. The animals were housed in standard cages and were maintained on a standard pelleted feed (Guinea Feed) and water *ad libitum*. Permission and approval for animal studies were obtained from the College of Health Sciences Animal Ethics Committee, University of Uyo.

### Parasite

A chloroquine sensitive strain of *Plasmodium berghei* (ANKA) was obtained from the National Institute of Medical Research (NIMR), Yaba Lagos, Nigeria and was maintained by sub-passage in mice while *Plasmodium falciparum* strains *Pf* 3D7 and *Pf* INDO were obtained from the International Center for Genetic Engineering and Biotechnology, New Delhi, India.

### Collection of plant materials

The fresh roots of *Alchornea laxiflora* were collected in August 2015 from a farmland in Nung Oku in Uruan LGA, Akwa Ibom State, Nigeria. The roots were identified and authenticated as *Alchornea laxiflora* by Dr. Margaret Bassey, a taxonomist in the Department of Botany and Ecological studies, University of Uyo, Uyo. Nigeria. Herbarium Specimen (FPHUU 536) was deposited at the Faculty of Pharmacy Herbarium, University of Uyo, Uyo.

### Extraction

The plant parts (root) were cut into smaller pieces, washed and air-dried on laboratory table for 2 weeks. The dried roots were pulverized using a pestle and mortar. The powdered root was macerated in 95% ethanol for 72 h. The liquid ethanol extract obtained by filtration was evaporated to dryness in a rotary evaporator 40 °C. The crude ethanol extract (20 g) was further partitioned successively with 2 L each of petroleum ether, dichloromethane, ethyl acetate and butanol to give the corresponding fractions of these solvents. The extract/fractions were stored in a refrigerator at 4 °C until used for experiment reported in this study.

### Phytochemical screening

Phytochemical screening of the crude root extract was carried out by employing standard procedures and tests (Trease & Evans 1989; Sofowora 1993), to reveal the presence of chemical constituents such as alkaloids, flavonoids, tannins, terpenes, saponins, anthraquinones, reducing sugars, cardiac glycosides and phlobatannins.

### Determination of acute toxicity in mice

This was done by determining the median lethal dose (LD_50_) of the extract using the method of Lorke’s (1983). This involved intraperitoneal (i.p.) administration of different doses of the extract (100–1000 mg/kg) to groups of five mice each. The animals were observed for manifestation of physical signs of toxicity such as writhing, decreased motor activity, decreased body/limb tone, decreased respiration and death. The number of deaths in each group within 24 h was recorded.

### Parasite inoculation

Each mouse used in the experiment was inoculated i.p. with 0.2 mL of infected blood containing about 1 × 10^7^*P. berghei* parasitized erythrocytes. The inoculum consisted of 5 × 10^7^*P. berghei* erythrocytes per mL. This was prepared by determining both the percentage parasitaemia and the erythrocytes count of the donor mouse and diluting the blood with isotonic saline in proportions indicated by both determinations (Odetola & Basir 1980).

### Drug administration

The drugs (chloroquine and pyrimethamine) and extract used in the antimalarial study were orally administered with the aid of a stainless metallic feeding cannula.

### Evaluation of *in vivo* antimalarial activity of ethanol crude root extract of *Alchornea laxiflora*

#### Evaluation of suppressive activity of the extract (four-day test)

Evaluation of the schizontocidal activity of the extract/fractions and chloroquine against early *Plasmodium berghei* infection in mice was done according to method described previously by Knight and Peters (1980). Forty-eight mice were infected with the parasite on the first day and randomly divided into eight groups of six mice each. The mice in group 1 were administered with the 75 mg/kg, the group 2, 150 mg/kg and group 3, 225 mg/kg of crude extract, while groups 4, 5 and 6 were respectively administered with 150 mg/kg of dichloromethane, ethyl acetate and *n*-butanol fractions. Group 7 was administered 5 mg/kg of chloroquine (positive control), and 10 mL/kg of distilled water to group 8 (negative control) for four consecutive days (D_0_–D_3_) between 8 am and 9 am. On the fifth day (D_4_), thin blood film was made from tail blood of each mouse and stained with Giemsa stain to reveal parasitized erythrocytes out of 500 in a random field of the microscope. The average percentage suppression of parasitaemia was calculated in comparison with the controls as follows:

$$\frac{\left(\mathrm{Average}\;\%\;\mathrm{parasitaemia}\;\mathrm{in}\;\mathrm{negative}\;\mathrm{control}-\mathrm{Average}\;\%\;\mathrm{parasitaemia}\;\mathrm{in}\;\mathrm{positive}\;\mathrm{groups}\right)}{\mathrm{Average}\%\text{parasitaemia }\mathrm{in}\;\mathrm{negative}\;\mathrm{control}}$$

The mean survival time (MST) of the mice was monitored in the different groups for 30 days.

#### Evaluation of prophylactic or repository activities of extract

The repository activity of the extract/fractions and pyrimethamine was assessed by using the method described by Peters (1965). Forty-eight mice were randomly divided into eight groups of six mice each. Groups 1–3 were administered with 75, 150 and 225 mg/kg/day of the extract, respectively. Groups 4, 5 and 6 were respectively administered with 150 mg/kg of dichloromethane, ethyl acetate or *n*-butanol. Groups 7 and 8 were administered with 1.2 mg/kg/day of pyrimethamine (positive control), and 10 mL/kg of distilled water (negative control), respectively. Administration of the extract/drug continued for three consecutive days (D_0_–D_2_). On the fourth day (D_3_), the mice were inoculated with *P. berghei berghei.* The parasitaemia level was assessed by blood smears 72 h later. The MST of the mice in each treatment group was determined over a period of 30 days.

#### Evaluation of curative activities of extract (Rane’s test)

Evaluation of the schizontocidal activity of the extract/fractions, and chloroquine in established infection was done according to the method of Ryley and Peters (1970). *P. berghei* was injected i.p. into 48 mice on the first day (D_0_). Seventy-two hours later (D_3_), infections were confirmed in the infected mice from Giemsa stained thin blood film made from tail blood of the mice and the parasitaemia level of each mouse determined. The mice were then divided randomly into eight groups of six mice each. Specific doses of the extract, 75, 150 and 225 mg/kg were orally administered respectively to mice in groups 1–3. Groups 4, 5 and 6 were respectively administered with 150 mg/kg of dichloromethane, ethyl acetate or *n*-butanol fractions. 5 mg/kg/day of chloroquine was administered to the group 7 (positive control) and group 8 was given 10 mL/kg of distilled water (negative control). The extract/fractions and drugs were administered once daily for five days. Giemsa stained thin smears were prepared from tail blood samples collected on each day of treatment to monitor parasitaemia level. The MST of the mice in each treatment group was determined over a period of 29 days (D_0_–D_28_).

### Evaluation of *in vitro* antiplasmodial activity

#### *In vitro* cultivation of plasmodium falciparum

CQ-sensitive strain 3D7 and CQ-resistant strain INDO of *Plasmodium falciparum* used in this study were *in vitro* blood stage cultures to test the antimalarial efficacy of the crude root extract and fractions. The culture was maintained at the Malaria Research Laboratory, International Center for Genetic Engineering and Biotechnology, New Delhi, India. *Plasmodium falciparum* culture was maintained according to the method described by Trager and Jensen (1976) with slight modifications. *Plasmodium falciparum* (3D7) cultures were maintained in fresh O + ve human erythrocytes suspended at 4% haematocrit in RPMI 1640 (Sigma, Darmstadt, Germany) containing 0.2% sodium bicarbonate, 0.5% albumax, 45 μg/L hypoxanthine and 50 μg/L gentamicin and incubated at 37 °C under a gas mixture of 5% O_2_, 5% CO_2_ and 90% N_2_. Daily, infected erythrocytes were transferred into fresh complete medium to propagate the culture. For *Plasmodium falciparum* (INDO strain) in culture medium, albumax was replaced by 10% pooled human serum.

#### Drug dilutions

Dimethyl sulphoxide (DMSO) was used to prepare the stock solutions of each plant extract and fraction as well as artemisinin, while water (Milli-Q grade) was used in the case of CQ stock solution. Culture medium was used to dilute the stock solutions to their required concentrations except CQ. The final solution of each stock was constituted to contain nontoxic concentration of DMSO (0.4%), which was found to be harmless to the parasite. Drugs, test plant extracts and fractions were then placed in 96-well flat bottom tissue culture grade plates.

#### *In vitro* antiplasmodial assays

The crude root extract and fractions of this plant were evaluated for their antiplasmodial activity against 3D7 and INDO strains of *Plasmodium falciparum*. For drug screening, SYBR green I-based fluorescence assay was set up as described previously (Smilkstein et al. 2004). Sorbitol synchronized parasites were incubated under normal culture conditions at 2% haematocrit and 1% parasitaemia in the absence or presence of increasing concentrations of plant extract and fractions. CQ and artemisinin were used as positive controls, while 0.4% DMSO was used as the negative control. After 48 h of incubation, 100 μL of SYBR Green I solution (0.2 μL of 10,000 × SYBR Green I (Invitrogen)/mL) in lysis buffer[Tris (20 mM; pH 7.5), EDTA (5 mM), saponin (0.008%, w/v) and Triton X-100 (0.08%, v/v)] was added to each well and mixed twice gently with multi-channel pipette and incubated in dark at 37 °C for 1 h. Fluorescence was measured with a Victor fluorescence multi-well plate reader (Perkin Elmer, Waltham, MA) with excitation and emission wavelength bands centred at 485 and 530 nm, respectively. The fluorescence counts were plotted against the drug concentration and the 50% inhibitory concentration (IC_50_) was determined by analysis of dose–response curves and IC_50_ estimator. Results were validated microscopically by examination of Giemsa stained smears of extract treated parasite cultures.

#### Cytotoxic activity on HeLa cells using MTT assay

The cytotoxic effects of extract and fractions on host cells were assessed by functional assay as described (Mosmann 1983) using HeLa cells cultured in RPMI containing 10% foetal bovine serum, 0.21% sodium bicarbonate (Sigma, Darmstadt, Germany) and 50 μg/mL gentamycin (complete medium). Briefly, cells (10^4^ cells/200 μL/well) were seeded into 96-well flat-bottom tissue culture plates in complete medium. Drug solutions were added after 24 h of seeding and incubated for 48 h in a humidified atmosphere at 37 °C and 5% CO_2_. DMSO (as positive inhibitor) was added at 10%. A stock solution (20 μL) of MTT (5 mg/mL in 1 × phosphate buffered saline) was added to each well, gently mixed and incubated for another 4 h. After spinning the plate at 1500 rpm for 5 min, supernatant was removed and 100 μL of DMSO (stop agent) was added. Formation of formazon was read on a microtiter plate reader (Versa max tunable multi-well plate reader) at 570 nm. The 50% cytotoxic concentration (IC_50_) of drug was determined by analysis of dose–response curves and IC_50_ estimator.

### Evaluation of analgesic potential of the extract

#### Acetic acid induced writhing in mice

Intraperitoneal injection of 3% acetic acid was used to induce writhings (abdominal constrictions consisting of the contraction of abdominal muscles together with the stretching of hindlimbs) according to the procedure described by Santos et al. (1994), Correa et al. (1996) and Nwafor et al. (2010). The animals were divided into eight groups of six mice each. Group 1 served as negative control and received 10 mL/kg of normal saline, while groups 2, 3 and 4 were pretreated with 75, 150 and 225 mg/kg doses of *A. laxiflora* root extract i.p., and groups 5, 6 and 7 were respectively administered 150 mg/kg of dichloromethane, ethyl acetate and *n*-butanol fractions. Group 8 received 100 mg/kg of acetyl salicylic acid (ASA). After 30 min, 0.2 mL of 2% acetic acid was administered i.p. The number of writhing movements was counted for 30 min. Antinociception (analgesia) was expressed as the reduction of the number of abdominal constrictions between control animals and mice pretreated with extracts.

#### Formalin-induced hind paw licking in mice

The procedure adopted was similar to that described by Hunskaar and Hole (1987), Gorski et al., (1993) and Okokon and Nwafor (2010). The animals were injected with 20 μL of 2.5% formalin solution (0.9% formaldehyde) made up in phosphate buffer solution (PBS concentration: NaCl 137 mM, KCl 2.7 mM and phosphate buffer, 10 mM) under the surface of the right hind paw. The amount of time spent licking the injected paw was timed and considered as indication of pain. Adult albino mice (20–25 g) of either sex randomized into eight groups of six mice each were used for the experiment. The mice were fasted for 24 h before used but allowed access to water. The animals in group 1 (negative control) received 10 mL/kg of normal saline, groups 2–4 received 75, 150 and 225 mg/kg doses of the extract respectively, while groups 5, 6 and 7 were respectively treated with dichloromethane, ethyl acetate and *n*-butanol fractions. Group 8 received 100 mg/kg of ASA 30 min before being challenged with buffered formalin. The responses were measured for 5 min (first phase) and 15–30 min (second phase) after formalin injection.

#### Thermally induced pain in mice

The effect of extract and fractions on hot plate induced pain was investigated in adult mice. The hot plate was used to measure the response latencies according to the method of Vaz et al. (1996) and Okokon and Nwafor (2010). In these experiments, the hot plate was maintained at 45 ± 1 °C, each animal was placed into a glass beaker of 50 cm diameter on the heated surface, and the time(s) between placement and shaking or licking of the paws or jumping was recorded as the index of response latency. An automatic 30 s cutoff was used to prevent tissue damage. The animals were randomly divided into eight groups of six mice each and fasted for 24 h but allowed access to water. Group 1 animal served as negative control and received 10 mL/kg of normal saline. Groups 2, 3 and 4 were pretreated i.p. with 75, 150 and 225 mg/kg doses of *A. laxiflora* root extract respectively, while groups 5, 6 and 7 were respectively treated with 150 mg/kg of dichloromethane, ethyl acetate and *n*-butanol fractions. Mice in group 8 received 100 mg/kg of ASA i.p., 30 min prior to the placement on the hot plate.

#### Gas chromatography–mass spectrometry analysis

Quantitative and qualitative data were determined by GC and GC–MS, respectively. The fraction was injected onto a Shimadzu GC-17A system (Kyoto, Japan), equipped with an AOC-20i autosampler and a split/splitless injector. The column used was an DB-5 (Optima-5), 30 m, 0.25 mm i.d., 0.25 μm df, coated with 5% diphenyl–95% polydimethylsiloxane. The oven temperature operated was programed as follows: 50 °C, held for 1 min, rising at 3 °C/min to 250 °C, held for 5 min, rising at 2 °C/min to 280 °C, held for 3 min. The injection temperature and volume were 250 °C and 1.0 μL, respectively. Injection mode, split; split ratio was 30:1. Carrier gas was nitrogen set at 30 cm/s linear velocity and inlet pressure of 99.8 kPa. Other operating parameters used included: detector temperature, 280 °C; hydrogen flow rate, 50 mL/min; air flow rate, 400 mL/min; make-up (H_2_/air), flow rate, 50 mL/min and sampling rate, 40 ms. Data were acquired by means of GC solution software (Shimadzu, Kyoto, Japan). Agilent 6890N GC was interfaced with a VG Analytical 70-250s double-focusing mass spectrometer. Helium was used as the carrier gas. The MS operating conditions were: ionization voltage 70 eV, ion source 250 °C. The GC was fitted with a 30 m × 0.32 mm fused capillary silica column coated with DB-5. The GC operating parameters were identical with those of GC analysis described above.

#### Identification of the compounds

The identification of compounds present in the active fraction of the plants’ extract was based on direct comparison of the retention times and mass spectral data with those for standard compounds, and by computer matching with the Wiley and Nist Libraries (Adams 2001; Setzer et al. 2007).

#### Statistical analysis

Data obtained from this work were analysed statistically using ANOVA (One-way) followed by a post test (Tukey–Kramer’s multiple comparison test). Differences between means were considered significant at 1% and 5% level of significance, that is *p* ≤ 0.01 and 0.05.

## Results

### Phytochemical screening

Phytochemical screening of the crude root extract revealed the presence of chemical constituents such as alkaloids, flavonoids, tannins, terpenes, saponins and cardiac glycosides.

### Determination of median lethal dose

The LD_50_ was calculated to be 748.33 mg/kg. The physical signs of toxicity included excitation, paw licking, increased respiratory rate, decreased motor activity, gasping and coma which was followed by death.

### Effect on suppressive activity of ethanol root extract/fraction of *Alchornea laxiflora*

The extract showed a dose-dependent chemosuppressive effect on the parasitaemia. These effects were statistically significant relative to the control (*p* < 0.05–0.001). The chemoinhibitory ranged from 25.87 to 44.05% (Table 1). Dichloromethane fraction had the highest activity with chemosuppression of 65.73%. The crude extract exhibited a MST range of 12.75 ± 0.47 to 18.25 ± 1.03 days, while DCM fraction had MST of 18.75 ± 0.62 days. However, the effect of the extract was weak compared to that of the standard drug, chloroquine, with a chemosuppression of 74.12% and MST of 24.25 ± 1.25 days (Table 1).

**Table 1.** Suppressive activities of root extract of *Alchornea laxiflora* (four-day test).

Drug/extract	Dose (mg/kg)	Parasitaemia	% chemosuppression	Mean survival time (days)
Distilled water	10 ml/kg	35.75 ± 3.06	–	12.75 ± 0.47
Extract	75	26.50 ± 1.50^b^	25.87	17.25 ± 0.75^b^
	150	24.50 ± 2.17^b^	31.46	17.72 ± 0.85^b^
	225	20.00 ± 3.74^a^	44.05	18.25 ± 1.03^b^
Dichloromethane	150	12.25 ± 2.20^c^	65.73	18.00 ± 0.40^b^
Ethyl acetate	150	14.25 ± 1.10^b^	60.13	17.25 ± 0.47^b^
*n*-Butanol	150	19.50 ± 2.63^a^	45.45	18.75 ± 0.62^b^
Chloroquine	5	9.25 ± 3.06^c^	74.12	24.25 ± 1.25^c^

Values are expressed as mean ± SEM.

Significance relative to control:

a *p* < 0.05;

b *p* < 0.01;

c *p* < 0.001, *n* = 6.

### Effect on repository activity of ethanol root extract/fractions of *Alchornea laxiflora*

The ethanol root extract of *Alchornea laxiflora* showed a dose-dependent chemosuppressive effect of 25.92–42.59% on the parasitaemia and MST range of 10.50 ± 0.50 to 18.50 ± 1.25 days during prophylactic studies. These effects were statistically significant relative to the control (*p* < 0.05–0.001). DCM fraction had the highest activity with chemosuppression of 55.55% and MST range of 19.25 ± 1.18 days. However, these effects were weak compared to that of the standard drug, pyrimethamine, with chemosuppression of 78.70% (Table 2).

**Table 2.** Repository/prophylactic activity of ethanol root extract of *Alchornea laxiflora* on *Plasmodium berghei* infection in mice.

Drug/extract	Dose (mg/kg)	Parasitaemia	% chemosuppression	Mean survival time (days)
Distilled water	10 ml/kg	27.00 ± 4.93	–	10.50 ± 0.50
Extract	75	20.0 ± 1.68^a^	25.92	16.35 ± 0.86^a^
	150	18.21 ± 0.81^c^	32.55	17.45 ± 0.75^a^
	225	15.50 ± 2.90^c^	42.59	18.50 ± 1.25^b^
Dichloromethane	150	12.00 ± 1.73	55.55	19.25 ± 1.18^c^
Ethyl acetate	150	14.75 ± 3.68	45.37	17.25 ± 1.29^b^
*n*-Butanol	150	17.00 ± 5.61	37.03	17.13 ± 1.32^b^
Pyrimethamine	1.2	5.75 ± 1.93^c^	78.70	25.0 ± 1.87^c^

Values are expressed as mean ± SEM.

Significance relative to control:

a *p* < 0.05;

b *p* < 0.01;

c *p* < 0.001, *n* = 6.

### Antiplasmodial effect of ethanol root extract of *Alchornea laxiflora* on established infection

The extract showed a dose-dependent schizonticidal effect on the parasitaemia. There were reductions in the percentage parasitaemia of the extract/fraction and chloroquine-treated groups compared to that of the control in which prominent increases were recorded. These reductions were statistically significant relative to the control (*p* < 0.0–0.001) (Table 3). The chemosuppression range of the extract treated groups was 50.0–76.52% on day 7. The crude extract also showed a significant (*p* < 0.05–0.001), dose-dependent MST (13.00 ± 0.40 to 19.00 ± 0.57 days) on established infection, and the MST value of dichloromethane fraction treated group was 19.25 ± 0.47 days. The highest dose of the extract as well as dichloromethane fraction (225 mg/kg) produced a chemosuppressive effects that were comparable to that of the standard drug, chloroquine (Table 3).

**Table 3.** Antiplasmodial activity of root extract of *Alchornea laxiflora* (curative test).

		Percentage mean reduction in parasitaemia per day					
Drug/extract	Dose (mg/kg)	3	4	5	7	% chemosuppression	Mean survivaltime (days)
Distilled water	10 ml/kg	31.50 ± 1.50	38.50 ± 1.12	43.25 ± 4.06	44.0 ± 2.61	–	13.00 ± 0.40
Extract	75	28.25 ± 2.83	31.50 ± 3.57	24.00 ± 5.80^a^	22.0 ± 2.85^c^	50.0	17.25 ± 1.04^a^
	150	33.00 ± 10.79	31.25 ± 1.98^b^	22.00 ± 6.40^c^	19.75 ± 4.15^b^	55.11	18.25 ± 0.85^b^
	225	27.50 ± 1.65	30.25 ± 1.29^c^	20.25 ± 2.98^c^	10.33 ± 2.52^c^	77.27	19.00 ± 0.57^b^
Dichloromethane	150	25.75 ± 7.14	28.0 ± 4.13	23.00 ± 2.49	11.25 ± 1.97^c^	74.43	19.25 ± 0.47^b^
Ethyl acetate	150	32.50 ± 6.66	32.75 ± 9.70	24.75 ± 5.66	14.25 ± 5.92^c^	67.61	18.75 ± 0.47^b^
*n*-Butanol	150	35.0 ± 2.83	32.75 ± 4.40	28.25 ± 2.83	22.50 ± 1.32^c^	48.86	18.00 ± 0.81^b^
Chloroquine	5	22.00 ± 3.02	22.0 ± 8.60^c^	19.25 ± 5.31^c^	10.75 ± 0.03^c^	75.56	30.00 ± 0.00^c^

Values are expressed as mean ± SEM.

Significant relative to control:

a *p* < 0.05;

b *p* < 0.01;

c *p* < 0.001, *n* = 6.

### *In vitro* antiplasmodial and cytotoxic activities

The results of the *in vitro* studies show that the root extract and fractions exerted antiplasmodial activity against chloroquine sensitive Pf 3D7 and resistant Pf INDO strains of *P. falciparum* (Table 4). The ethyl acetate fraction was found to exhibit moderate activity against both strains of *P. falciparum* with IC_50_ value of 38.44 ± 0.89 μg/mL (Pf 3D7) and 40.17 ± 0.78 μg/mL (Pf INDO). The potency order was ethyl acetate > crude extract > dichloromethane > petroleum ether. The crude extract and fractions were not cytotoxic to HeLa cell lines tested with TC_50_ values >100 μg/mL.

**Table 4.** *In vitro* antiplasmodial activities of crude root extract and fractions of *A. laxiflora*.

Crude extract/fraction	IC_50_ (μg/ml)*Pf* 3D7	IC_50_ (μg/ml)*Pf* INDO	CytotoxicityHeLa cells IC_50_(μg/ml)
Crude extract	52.73 ± 2.26	56.71 ± 3.43	>100
Pet. ether	81.20 ± 2.34	90.24 ± 3.38	>100
Dichloromethane	72.72 ± 1.14	73.48 ± 2.35	>100
Ethyl acetate	38.44 ± 0.89	40.17 ± 0.78	>100
Butanol	>100	98.99 ± 1.53	>100
Aqueous	>100	>100	>100
Chloroquine	0.021	0.258	–
Artemisinin	0.0045	0.0045	–

### Effect of ethanol root extract of *A. laxiflora* on acetic acid-induced writhing in mice

The administration of *A. laxiflora* extract (75, 150 and 225 mg/kg) demonstrated a dose-dependent reduction in acetic acid-induced writhing in mice. The reductions were statistically significant (*p* < 0.05–0.001) relative to control during the first 20 min of the experiment. The dichloromethane and ethyl acetate fractions exerted activities that were comparable to that of the standard drug, ASA (Table 4).

### Effect of ethanol root extract of *A. laxiflora* on formalin induced paw licking in mice

The administration of *A. laxiflora* extract (75, 150 and 225 mg/kg) demonstrated a dose-dependent reduction in formalin-induced hind paw licking in mice. The reductions were statistically significant (*p* < 0.05–0.001) relative to control and were persistent from 10 to 25 min of the experiment. The ethyl acetate fraction exerted the highest activity which was comparable to that of the standard drug, ASA, 100 mg/kg. The effect of the crude extract was diminished in the last 5 min of the 30 min duration of the experiment (Table 5).

**Table 5.** Effect of *Alchornea laxiflora* root extract on acetic acid induced writhing in mice.

		Time intervals (h)						
Treatments	Dose (mg/kg)	5	10	15	20	25	30	Total
Control	10 ml/kg	9.25 ± 0.85	10.75 ± 0.75	14.00 ± 1.78	22.25 ± 0.85	13.50 ± 0.64	14.50 ± 2.53	84.25 ± 7.40
Extract	75	6.75 ± 0.62	12.75 ± 0.62	12.25 ± 2.09^b^	17.0 ± 1.35^b^	13.25 ± 2.05	10.25 ± 1.43	72.25 ± 8.16^a^
	150	4.50 ± 0.64	5.25 ± 1.03^a^	14.00 ± 1.68^c^	15.0 ± 2.34^c^	18.25 ± 2.78^a^	22.25 ± 1.65^a^	79.25 ± 8.45
	225	4.75 ± 0.47^c^	5.00 ± 0.91^b^	11.50 ± 1.55^c^	20.00 ± 2.67^c^	13.25 ± 1.54	15.50 ± 1.32	70.00 ± 8.46^a^
Dichloromethane fraction	150	1.00 ± 0.57^c^	2.50 ± 1.19^b^	1.50 ± 0.95^b^	4.75 ± 0.62^c^	1.75 ± 0.85^c^	1.25 ± 0.62^c^	12.75 ± 4.80^c^
Ethyl acetate fraction	150	4.00 ± 1.08^b^	2.75 ± 1.25^b^	3.25 ± 1.25^b^	2.00 ± 1.08^c^	1.50 ± 0.95^c^	1.00 ± 0.70^c^	15.25 ± 6.31^c^
*n*-Butanol fraction	150	3.00 ± 0.91^c^	3.00 ± 1.91^b^	8.25 ± 3.27	10.50 ± 2.63^b^	10.00 ± 1.41	8.00 ± 1.41	42.75 ± 11.54^c^
ASA	100	2.25 ± 0.31^a^	2.50 ± 0.19^b^	4.75 ± 0.49^a^	2.75 ± 0.10^c^	1.50 ± 0.50b	0.50 ± 0.15^c^	14.25 ± 1.74^c^

Data are expressed as mean ± SEM.

Significant at

a *p* < 0.05,

b *p* < 0.01,

c *p* < 0.001 when compared to control *n* = 6.

### Effect of ethanol root extract of *A. laxiflora* on thermally-induced pain in mice

The extract (75, 150 and 225 mg/kg) exhibited a dose-dependent effect on thermally-induced pain in mice. These inhibitions were statistically significant (*p* < 0.05–0.001) relative to the control. The ethyl acetate fraction exerted the highest activity which was weak compared to that of the standard drug, ASA (100 mg/kg) (Table 6).

**Table 6.** Effect of *Alchornea laxiflora* root extract on formalin hind paw licking in mice.

		Time intervals (h)						
Treatments	Dose (mg/kg)	5	10	15	20	25	30	TOTAL
Control	10 ml/kg	23.50 ± 0.86	23.0 ± 0.70	20.50 ± 1.44	13.50 ± 0.28	14.25 ± 0.25	11.50 ± 1.50	106.25 ± 5.03
Extract	75	17.25 ± 2.17	2.75 ± 1.37^c^	2.50 ± 0.28^b^	2.75 ± 0.47^b^	8.75 ± 1.49^b^	15.50 ± 1.44	49.50 ± 7.22^c^
	150	21.50 ± 1.84	1.25 ± 0.94^c^	2.50 ± 0.28^c^	0.25 ± 0.25^c^	5.25 ± 0.47^c^	12.50 ± 0.95	43.25 ± 4.73^c^
	225	25.75 ± 4.07^c^	1.50 ± 0.86^b^	1.50 ± 0.95^c^	4.00 ± 1.82^c^	7.50 ± 0.86^b^	13.50 ± 1.50	53.75 ± 10.06^c^
Dichloromethane fraction	150	10.75 ± 1.88	2.75 ± 0.75^c^	0.75 ± 0.47^c^	1.25 ± 0.75^c^	5.25 ± 1.03^c^	10.00 ± 3.94	30.75 ± 8.82^c^
Ethyl acetate fraction	150	9.25 ± 1.49^a^	1.50 ± 0.95^c^	0.75 ± 0.47^c^	2.50 ± 1.04^c^	0.25 ± 0.01^c^	0.00 ± 0.00^c^	14.25 ± 3.96^c^
*n*-Butanol fraction	150	21.75 ± 2.47^c^	1.75 ± 0.85^c^	2.75 ± 1.79^c^	7.00 ± 1.73^b^	11.25 ± 3.94	17.25 ± 0.62	61.75 ± 11.40^c^
ASA	100	15.75 ± 1.03^a^	3.50 ± 0.64^b^	2.00 ± 0.40^c^	2.00 ± 0.70^c^	1.50 ± 0.28^c^	2.50 ± 0.28^c^	27.25 ± 3.33^c^

Data are expressed as mean ± SEM.

Significant at

a *p* < 0.05,

b *p* < 0.01,

c *p* < 0.001 when compared to control *n* = 6.

### GCMS analysis

The GCMS analysis of the ethyl acetate fraction of *A. laxiflora* root revealed the presence of 43 bioactive compounds with major and minor ones as represented in Table 8.

**Table 7.** Effect of *Alchornea laxiflora* root extract on hot plate test.

Treatments	Dose (mg/kg)	Reactiontime (s)(mean ± SEM)	% inhibition
Control	10ml/kg	11.33 ± 0.52	–
Extract	75	16.70 ± 1.18^a^	47.39
	150	17.68 ± 1.27^a^	56.04
	225	19.09 ± 1.05^c^	68.49
Dichloromethane fraction	150	17.97 ± 0.80^b^	58.60
Ethyl acetate fraction	150	18.51 ± 0.75^b^	63.37
*n*-Butanol fraction	150	14.11 ± 0.22^c^	24.53
ASA	100	23.64 ± 1.69^c^	108.64

Data are expressed as mean ± SEM.

Significant at

a *p* < 0.05,

b *p* < 0.01,

c *p* < 0.001 when compared to control *n* = 6.

**Table 8.** GCMS profile of ethyl acetate fraction of *Alchornea laxiflora* root.

Peak	RT	Compound name	Formula	Mol. mass
1	6.957	Propanoic acid, 3-(trimethylsilyl)-, ethyl ester	C_8_H_18_O_2_Si	174
2	8.358	2-Furancarboxylic acid, trimethylsilyl ester	C_8_H_12_O_3_Si	184
3	11.277	Cyclopropenoic acid,1-trimethylsilyl,-2-(2-methylpropen-1-yl), methyl ester	C_12_H_20_O_2_Si	224
4	11.955	2H-Pyran-2-one, 5,6-dihydro-6-pentyl-	C_10_H_16_O_2_	168
5	12.476	1,2,4-Cyclopentanetrione, 3-butyl-	C_9_H_12_O_3_	168
6	14.457	Phenol, 3-[(trimethylsilyl)oxy]-	C_9_H_14_O_2_Si	182
7	14.918	1-Tetradecene	C_14_H_28_	196
8	15.110	Octadecane, 1-bromo-	C_18_H_37_Br	332
9	16.299	2-Butenoic acid, 2-methoxy-3-methyl-, methyl ester	C_7_H_12_O_3_	144
10	16.627	Benzoic acid, 3-acetyloxy-, trimethylsilyl ester	C_12_H_16_O_4_Si	252
11	17.605	Cyclopropanecarboxylic acid, 2,2-dimethyl-3-cis-(2-methyl-3-buten-2-yl)-	C_11_H_18_O_2_	182
12	18.624	2H-Pyran-2-one, tetrahydro-4-hydroxy-6-pentyl-	C_10_H_18_O_3_	186
13	18.905	3-{[*tert*-Butyl(dimethyl)silyl]oxy}butanal	C_10_H_22_O_2_Si	202
14	19.833	1-Hexadecene	C_16_H_32_	224
15	19.910	Dodecanoic acid, ethyl ester	C_14_H_28_O_2_	228
16	24.291	1-Octadecene	C_18_H_36_	252
17	24.348	Tetradecanoic acid, ethyl ester	C_16_H_32_O_2_	256
18	26.421	Pentadecanoic acid, ethyl ester	C_17_H_34_O_2_	270
19	27.137	Hexadecanoic acid, methyl ester	C_17_H_34_O_2_	270
20	28.777	1-Heptacosanol	C_27_H_56_O	396
21	28.964	Hexadecanoic acid, ethyl ester	C_18_H_36_O_2_	284
22	30.511	Hexadecanoic acid, trimethylsilyl ester	C_19_H_40_O_2_Si	328
23	31.920	Octadecanoic acid, ethyl ester	C_20_H_40_O_2_	312
24	32.103	9-Octadecenoic acid (Z)-, methyl ester	C_19_H_36_O_2_	296
25	33.665	*n*-Propyl 9,12-octadecadienoate	C_21_H_38_O_2_	322
26	33.776	9-Octadecenoic acid (z)-, ethyl ester	C_20_H_38_O_2_	310
27	33.986	Octadecanoic acid	C_18_H_36_O_2_	284
28	34.204	Octadecanoic acid, ethyl ester	C_20_H_40_O_2_	312
29	34.632	*trans*-9-Octadecenoic acid, trimethylsilyl ester	C_21_H_42_O_2_Si	354
30	34.980	9,12-Octadecadienoic acid (*Z*, *Z*)-	C_18_H_32_O_2_	280
31	37.069	Octadecanoic acid, ethyl ester	C_20_H_40_O_2_	312
32	38.101	Zeranol	C_18_H_26_O_5_	322
33	38.927	Benzeneacetic acid, alpha-[(trimethylsilyl)oxy]-,	C_14_H_24_O_3_Si_2_	296
34	39.153	Capsaicin	C_18_H_27_NO_3_	305
35	39.368	Dihydrocapsaicin	C_18_H_29_NO_3_	307
36	39.791	2-Hydroxy-3-methoxybenzaldehyde, trimethylsilyl ether	C_11_H_16_O_3_Si	224
37	40.192	1,1′-Biphenyl-3,4,4′-trimethoxy-6′-formyl-	C_16_H_16_O_4_	272
38	40.724	11-*cis*-Octadecenoic acid 1tms	C_21_H_42_O_2_Si	354
39	41.279	Ethyl tetracosanoate	C_26_H_52_O_2_	396
40	41.719	Squalene	C_30_H_50_	410
41	44.151	Octadecanoic acid, ethyl ester	C_20_H_40_O_2_	312
42	44.362	Benzene, (2-ethyl-4-methyl-1,3-pentadienyl)-, €-	C_14_H_18_	186
43	57.636	Cholest-4-en-3-one	C_27_H_44_O	384

## Discussion

The root of *A. laxiflora* is used in Ibibio folkloric medicine as malaria remedy and this work was designed to confirm and authentic its antiplasmodial potential in order to provide scientific basis for its usage as antimalarial plant.

In this work, LD_50_ was determined to be 748.33 mg/kg, portraying the extract to be slightly toxic (Homburger 1989) and the doses (75, 150 and 300 mg/kg) employed in this study were relatively safe.

The antiplasmodial activity of root extract and fractions of *Alchornea laxiflora* was investigated for antimalarial activity against rodent malaria parasite, *P. berghei*, infection in mice using standard *in vivo* models. It was found that the extract significantly reduced the parasitaemia in prophylactic, suppressive and curative models in a dose-dependent fashion and the dichloromethane and ethyl acetate fractions were found to demonstrate considerable activities confirming the antimalarial potential of this extract. The extract and fractions also prolonged the MST of the mice suggesting that they were able to offer certain degree of protection to the mice. This activity could have resulted from plasmodicidal or plasmodistatic activity of the extract and fractions. These results validate the use of the root decoctions as malarial remedy.

Further work was carried out to evaluate the activities of the extract and fractions against human malaria parasite, *P. falciparum*. The *in vitro* antiplasmodial study on the root extract and fractions of *A. laxiflora* carried out against chloroquine sensitive (Pf 3D7) and resistant (Pf INDO) strains of human malaria parasite, *P. falciparum*, further showed that the root extract and fractions possess moderate antiplasmodial activity against the parasites. Ethyl acetate fraction was found to exert the most pronounced activity probably suggesting the localization of the active compounds in this fraction. The root extract and fractions were observed to be active also against the chloroquine resistant strain (Pf INDO). This suggests that the root extract could be effective in the treatment of resistant malaria infection. However, dichloromethane fraction was outstandingly more active during the *in vivo* testing as compared to *in vitro* activity. This suggests the involvement of immunostimulating activity which may be due to the phytochemical constituents in this fraction. Tannins, squalene and fatty acids such as linoleic acids have been reported to possess immune-stimulating properties (Kolodziej et al. 2001; Chakrabarti et al. 2012; Kumaradevan et al. 2015). These compounds are found to be present in this fraction and extract may be responsible for the suggested immunostimulatory effect.

The phytochemical screening and GCMS analysis of the crude extract and ethyl acetate fraction have revealed the present of some pharmacologically active compounds such as flavonoids, alkaloids, terpenes, triterpenes like squalene, tannins, phenolics and polyunsaturated fatty acids (PUFAs) among others. These compounds are likely to be responsible for the observed activities of the extract and fractions. Some secondary metabolites of plants such as alkaloids, flavonoids and triterpenoids have been reported previously to have antiplasmodial properties (Kirby et al. 1989; Philipson & Wright 1991; Christensen & Kharazmi 2001).

Polyunsaturated fatty acids such as hexadecanoic acid, methyl ester, 9,12-octadecadienoic acid methyl ester (linoleic acid), 9,12,15-octadecatrienoic acid, methyl ester (linoleic acid), 9-octadecenoic acid (*Z*)-2-hydroxyethyl ester and eicosanoic acid, 2-(acetyloxy)-1-[(acetyloxy)methyl]ethyl ester have been found in the active antiplasmodial fraction. These PUFAs above have been implicated in antiplasmodial activity and this activity has been reported to increase with the degree of unsaturation (Kumaratilake et al. 1992; Krugliak et al. 1995; Suksamrarn et al. 2005; Attioua et al. 2007; Melariri et al. 2011, 2012). Flavonoids such as quercetin, quercetrin, rutin, taxifolin and quercetin 3,4-diacetate have also been isolated from ethyl acetate leaf fraction of *A. laxiflora* (Ogundipe et al. 2001; Adeloye et al. 2005). These compounds are likely to be present in the root probably in varying quantity. Quercetin and its derivatives as well as rutin have been shown to possess significant antiplasmodial activity against chloroquine sensitive and resistant strains of *P. falciparum* (Attioua et al. 2011; Ganesh et al. 2012; Ezenyi et al. 2014). Rutin has been reported to possess IC_50_ of 3.53 ± 13.34 μM against 3D7 and 15.00 μM against K1 (Attioua et al. 2011). Farombi et al. (2003) reported the presence of terpenoids compounds in the root and GCMS analysis of ethyl acetate fraction further revealed the presence of squalene, a triterpene and active antioxidant compound (Kohno et al. 1995). These compounds mentioned above to be present in the extract and active fraction maybe responsible for the observed antiplasmodial activities. Antioxidant property of quercetin has been suggested to be responsible for its antiplasmodial activity (Cimanga et al. 2009; Ganesh et al. 2012), as elevated free radical levels are common features of malaria disease and are implicated in severe malaria complications. This could be one of the mechanisms of action of this extract. Other mechanisms of antiplasmodial activity have been proposed for flavonoids besides the antioxidant activity. Flavonoids are known to exert antiplasmodial activity by chelating with nucleic acid base pairing of the parasite (Lui et al. 1992), thereby producing plasmodicidal effect. Other modes of action include modulation of host immunity to tackle disease and inhibition of plasmodial enoyl-ACP reductase (FAB I enzyme) – a key regulator of type II fatty synthases (FAS-II) in *P. falciparum* (Kirmizibekmez et al. 2004; Teffo et al. 2010). Flavonoids may also bind parasite’s serinethreonine kinase with high affinity and affect its development (Ferreira et al. 2010). The root extract may be acting through one of these mechanisms.

These compounds (flavonoids) present in this plant extract and in particular, ethyl acetate fraction may in part have contributed to the plasmodicidal activity of this extract/fraction and therefore explained the mechanism of antiplasmodial effect of the extract.

Phytochemical compounds such as terpenes and their derivatives as well as alkaloids which have been found to be present in this plant, have been reported previously to contribute to antiplasmodial activity of many plants (Philipson & Wright 1991; Christensen & Kharazmi 2001). These could have also contributed to the antiplasmodial activity of this extract.

The root extract and fractions were found to possess analgesic activity against acetic acid-induced writhing, formalin induced hind paw licking and thermal induced pains with the ethyl acetate and chloroform fractions exhibiting prominent activity.

Acetic acid causes inflammatory pain by inducing capillary permeability (Amico-Roxas et al. 1984; Nwafor et al. 2007), and in part through local peritoneal receptors from peritoneal fluid concentration of PGE_2_ and PGF_2α_ (Deraedt et al. 1980; Bentley et al. 1983). The acetic acid-induced abdominal writhing is a visceral pain model in which the processor releases arachidonic acid via cyclooxygenase, and prostaglandin biosynthesis plays a role in the nociceptive mechanism (Franzotti et al. 2002). It is used to distinguish between central and peripheral pain. These results suggested that the extract may be exerting its action partly through the lipoxygenase and/or cyclooxygenase system.

The organic acid has also been suggested to induce the release of endogenous mediators indirectly, which stimulates the nociceptive neurons that are sensitive to NSAIDs and narcotics (Adzu et al. 2003). The inhibition of acetic acid-induced writhing by the extract at all the doses suggests antinociceptive effect which might have resulted from the inhibition of the synthesis of arachidonic acid metabolites.

Formalin-induced pains involve two different types which are in phases; neurogenic and inflammatory pains (Vaz et al. 1996, 1997) and measure both centrally and peripherally mediated activities that are characteristics of biphasic pain response. The first phase (0–5 min), named neurogenic phase resulted from chemical stimulation that provoked the release of bradykinin and substance P while the second and late phase initiated after 15–30 min of formalin injection resulted in the release of inflammatory mediators such as histamine and prostaglandin (Wibool et al. 2008; Yi et al. 2008). The injection of formalin has been reported to cause an immediate and intense increase in the spontaneous activity of C fibre afferent and evokes a distinct quantifiable behaviour indicative of pain demonstrated in paw licking by the animals (Heapy et al. 1987). The first phase of formalin-induced hind paw licking is selective for centrally acting analgesics such as morphine (Berken et al. 1991), while the late phase of formalin-induced hind paw licking is peripherally mediated. Analgesic (nociceptive) receptors mediate both the neurogenic and non-neurogenic pains (Lembeck & Holzer 1979). The extract ability to inhibit both phases of formalin-induced paw licking suggests its central and peripheral activities as well as its ability to inhibit bradykinins, substance P, histamine and prostaglandins which are mediators in these pains.

The study also shows that the extract significantly delayed the reaction time of thermally-induced (hot plate) test. This model is selective for centrally acting analgesics and indicates narcotic involvement (Turner 1995) with opioid receptors. This finding further confirms the central analgesic action of the extract.

Some terpenes, flavonoids and polyphenolic compounds have also been revealed by GCMS analysis to be present in the plant extract. Flavonoids are known anti-inflammatory compounds acting through inhibition of the cyclo-oxygenase pathway (Liang et al. 1999). Some flavonoids are reported to block both the cyclooxygenase and lipoxygenase pathways of the arachidonate cascade at relatively high concentrations, while at lower concentrations they only block lipoxygenase pathway (Carlo Di et al. 1999). Some flavonoids exert their antinociception via opioid receptor activation activity (Suh et al. 1996; Rajendran et al. 2000; Otuki et al. 2005). Flavonoids also exhibit inhibitory effects against phospholipase A_2_ and phospholipase C (Middleton et al. 2000), and cyclooxygenase and/or lipoxygenase pathways (Robak et al. 1998). One of these mechanisms could likely be the mode of analgesic action of these extract and fractions.

Triterpenes have also been implicated in analgesic activity of plants (Liu 1995; Krogh et al. 1999; Tapondjou et al. 2003; Maia et al. 2006). Ursolic acid is a selective inhibitor of cyclooxygenase-2 (Ringbom et al. 1998). Oleanolic acid is known to exert its analgesic action through an opioid mechanism, and possibly, a modulatory influence on vanilloiod receptors (Maia et al. 2006). Squalene, a triterpene and strong antioxidant found in this plant may have contributed to the observed analgesic effect.

Moreso, capsaicin and dihydrocapsaicin, alkaloids, have been revealed to be present in the ethyl acetate fraction. Capsaicin is efficacious in neuropathic pain and its analgesic activity has been reported by Jolayemi and Ojewole (2014) to be effective in inhibiting acetic acid induced writhing and hot plate induced pain. Capsaicin, a transient receptor potential vanilloid 1 (TRPV1) agonist, and its metabolites are natural ligands for TRPV1 receptors, which are ion channel-type receptors found on sensory neurons. Capsaicin displays analgesic activity, potentially by inducing desensitization of TRPV1 receptors after activation (Smith & Brooks 2014).

The above extract has been reported to exhibit analgesic activity. The presence of these compounds (polyphenolics, flavonoids and triterpenes) in this plant may have accounted for these activities and may in part explain the mechanisms of its actions in this study.

## Conclusions

The results obtained in this study indicated that the root of *Alchornea laxiflora* plant possesses significant antimalarial and antiplasmodial activities against chloroquine sensitive and resistant strains of *P. falciparum* and also has analgesic action. These findings justify and confirm the usage of this plant in the treatment of malaria and related symptoms. Further studies on the extract and ethyl acetate fraction are necessary to isolate, characterize and identify the active compound which could serve as a useful agent against resistant malaria infections.
